# Underlying determinants of maternal mortality in a rural South African population with high HIV prevalence (2000–2014): A population-based cohort analysis

**DOI:** 10.1371/journal.pone.0203830

**Published:** 2018-09-13

**Authors:** B. Tlou

**Affiliations:** Discipline of Public Health Medicine, School of Nursing and Public Health, University of KwaZulu-Natal, Durban, South Africa; Stellenbosch University, SOUTH AFRICA

## Abstract

**Introduction:**

Maternal mortality is one of the significant health indicators of any country and it’s a frequent subject in many global heath discussions. Even though the global trends have shown a decrease on maternal mortality, many countries in sub-Saharan Africa failed to achieve the MDG 5 target in 2015.There is no specific single solution for reducing maternal mortality but there is unanimity that a reliable health system with skilled personal is vital for addressing maternal mortality. This study therefore seeks to identify the risk factors for maternal mortality in typical rural sub-Saharan African countries.

**Method:**

A longitudinal population based cohort study was conducted using data from 2000–2014 in Africa Health Research Institute (AHRI).The Cox regression method was used to assess the influence of selected risk factors using the Mosley-Chen model on maternal mortality. A total of 20701 women aged 15–49 years were included in the study.

**Results:**

The study found 212 maternal deaths from 32,620 live births with a maternal mortality ratio (MMR) of 650 per 100,000 live births. The main causes of death were Communicable diseases (38.2%), Aids and TB (31%) and Unknown causes (11.8%). An increased risk of death was identified on, poor wealth index (HR 3.92[1.01, 15.3]), period of death 2000–2006(HR32.1 [3.79, 71.5]) and number of deliveries (6.76[2.70, 16.9]) were associated with a high risk of maternal mortality after adjusting for other independent variables included in the study.

**Conclusion:**

Socio-economic status, number of deliveries and period of death were found to be associated with maternal death in rural South Africa.

## Introduction

According to World Health Organisation, maternal mortality is defined as “the death of a woman while pregnant or within 42 days of termination of pregnancy, irrespective of the duration and site of the pregnancy, from any cause related to or aggravated by the pregnancy or its management but not from accidental or incidental causes [1]”. The world health organisation (WHO) global maternal mortality statistics have shown that maternal mortality has decreased by 45% even though approximately 33 women are still dying per hour [2]. Sadly, even if the global trends are decreasing, the decline is not homogeneous distributed in the sub-Saharan region. There is still quite a lot third world countries, which still has high maternal mortality rates, and these include Sierra Leone, Somalia, Liberia and Chad [3]. Statistics have shown that South Africa had a maternal mortality rate of 138 deaths per 100 000 live births in 2015 [4].There has been contradicting statistics for maternal mortality in rural South Africa based on the findings from different studies.

Some of the areas and regions around the world have progressed on maternal mortality reduction even though the figures remain repugnantly huge in sub-Saharan Africa. Sub-Saharan Africa still experiences high inaccessibility of antenatal care, lack of skilled personal to attend to birth complications and huge fertility rates. The main leading causes of maternal mortality are complications in pregnancy, bleeding after birth, sepsis, unsafe abortion and obstructed labour. Lack of substantial advancement on maternal mortality has led to the inability of achieving MDG5 of a three quarter decline on maternal mortality and unfortunately, South Africa is one of them.

Maternal mortality is a rare event in most rural sub-Saharan countries largely due to the inadequacy of the huge longitudinal population data sets, which makes it challenging to monitor the factors influencing maternal mortality particularly in rural areas. As a result, individual risk factors for maternal mortality are regularly unknown and often vary over time, which makes it difficult to make conclusions about the underlying risk factors of maternal mortality. In most cases, maternal mortality is often misclassified as being caused by other external factors besides pregnancy related complications particularly in most sub-Saharan African countries [5, 6, 7]. Even though there has been significant progress on the decline of maternal mortality there is still a huge doubt whether the 2030 sustainable development goals will be achieved hence the need to identify the maternal mortality risk factors particularly in rural sub-Saharan Africa, based on the broadness of the SDGs as compared to the fifth 2015 millennium developmental goal (MDG5) [8].

In addition, maternal mortality is a health indicator that displays broad disparities both between and within communities’ particularly urban and rural communities. In order to reduce maternal fatalities from pregnant related complications, it is very important to explicitly define the risk factors that influences the complications. As already highlighted previously that maternal mortality is a rare event, the availability of the longitudinal data sets in rural South Africa enables researches to study the possible risk factors for maternal mortality. The inadequacy of robust and complete information on maternal deaths often hinders policy interventions and implementations to address the dilemma. Thus, in order for policy makers to guide interventions on maternal mortality there is a fundamental need to understand the risk factors associated with maternal mortality.

## Methods

### Study setting

**Fig 1:**
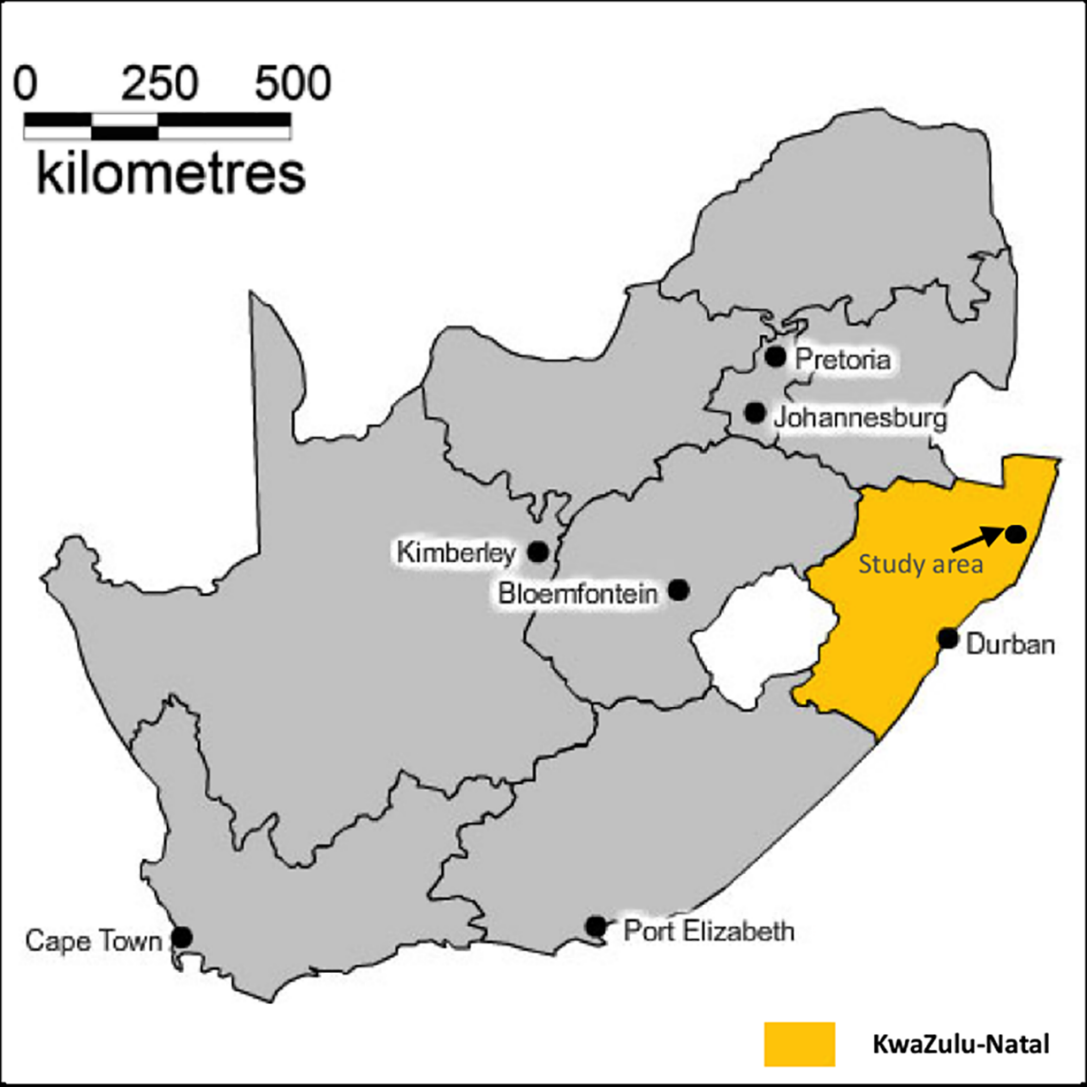
Location of the Africa Health Research Institute (AHRI) study area in KwaZulu-Natal Province, South Africa.

This study was conducted in AHRI (Africa Health Research Institute) located in northern rural KwaZulu Natal, South Africa (Fig 1). The study site is made up of approximately 11 000 households constituting a population of about 90 000 individuals within a geographical area of 438 square kilometers [9, 10, 11]. The AHRI collects demographic characteristics (births, deaths and migrations) for households and individuals residing in the study area three times a year [12]. The research institute also conducts annual population based HIV surveillance for individuals aged 15myears or older using trained field workers [13]. The study area has a high prevalence of HIV and unemployment even though the incidence has been declining for the past recent years [14]. Ethical approval for this study was received from the Biomedical Research Ethics Committee (BREC) of the University of KwaZulu-Natal (BE 169/15). All the data were fully anonymized before I accessed them and the ethics committee waived the requirement for informed consent since this was a retrospective study.

### Statistical analysis

Maternal mortality was determined using verbal autopsy and defined using the WHO definition as” the death of a woman while pregnant or within 42 days of termination of pregnancy, irrespective of the duration and site of the pregnancy, from any cause related to or aggravated by the pregnancy or its management, but not from accidental or incidental causes.” The study used the Mosley Chen and Meade models [15] to identify maternal mortality risk factors classified at individual, household and community level. The wealth index for individual households was calculated using the Principal component analysis (PCA).The Cox proportional hazards regression was used to assess the risk factors for maternal mortality examined concurrently to survival time, with the hazard rate used as the measure of effect. The study observed time t for each participant from the time of birth until death or date of migration for the emigrated and in-migrated study participants. The supremum test was used to assess the proportional hazard assumption. Collinearity between risk factors was assessed using the variance inflation factor and tolerance values. Data analysis was conducted using STATA software version 14 [16].

## Results

This study used longitudinal data collected from 2000 to 2014, with 32,620 live births recorded and 212 maternal deaths giving a maternal mortality ratio of approximately 650 per 100,000 live births. Approximately 54% of the women refused to test for HIV during the stipulated study period. In addition, results from population viral load surveys in this area show that the viral suppression level among females has increased from 28.2% in 2000 to 44.7% in 2014. Between the years 2000 and 2014 the proportion of women with HIV who were on antiretroviral therapy (ART) increased from 29% to 49%. Tuberculosis was the main the leading cause of death among HIV/Aids patients, on ART treatment.Approximately 8% of the study participants were HIV positive and the majority of them (78.5%) had primary education or none. Most maternal deaths occurred during the years 2000–2006, general people in the study site had a poor socio-economic status, and about 46% of the population resided in rural areas. The average parity was two children per woman and most women (22.2%) were within the 20–24 years age group. Table 1 below summarises the demographic characteristics of women in the study from 2000–2014.

**Table 1:** Demographic characteristics of women of child bearing age (15–49) in Africa Health Research Institute, South Africa (2000–2014).

Variable	Categories of variable	Percentage
**Maternal death**	Yes	1.1
	No	98.9
**HIV Status**	Positive	8.4
	Negative	91.6
**Area**	Rural	45.8
	Peri-urban	37.9
	Urban	16.3
**Number of deliveries**	None	98.8
	Singletons	1.1
	Multiple deliveries	0.1
**Age**	15–19	17.6
	20–24	22.2
	25–29	20.1
	30–34	15.9
	35–39	10.9
	40–44	7.7
	45–49	5.5
**Year of Maternal death**	2000–2006	56.3
	2007–2014	43.7
**Distance to the nearest clinic**	Less than 10 km	78.5
	Greater than or equal to 10 km	21.5
**Education**	Primary and less	78.5
	Secondary and more	21.5
**Source of drinking water**	Piped water	54.5
	Borehole	17.5
	Well	11.3
	Surface	11.9
	Other(Tanks)	4.8
**Wealth Index**	Poor	42
	Middle	19.2
	Rich	38.8

The study also assessed the influence of selected factors from the Mosley Chen and Meade models on maternal mortality. Firstly, each factor was associated with maternal mortality independently and only those factors, which were significantly associated with maternal mortality, were put into the multivariable model. Only wealth index, source of drinking water, education level, period of death, area and number of deliveries were individual associated with maternal mortality. After adjusting for other study variables, poor wealth index (HR 3.92[1.01, 15.3]), period of death 2000–2006(HR32.1 [3.79, 71.5]) and number of deliveries (6.76[2.70, 16.9]) were associated with a high risk of maternal mortality as shown in Table 2.

**Table 2:** Univariate and multivariate hazard ratios for risk factors associated with maternal mortality, Africa Health Research Institute (South Africa) using Cox proportional hazards regression.

		Univariate			Multivariable		
Explanatory variables	Categories of explanatory variables	Hazard ratio	95%Confidence Interval	p- value	Adjusted Hazard Ratio	95%Confidence interval	p-value
**Wealth Index**	Poor	2.86	1.48–5.55	<0.002	3.92	1.01–15.3	0.049
	Middle	2.02	0.89–4.57	0.092	1.52	0.32–7.34	0.601
	Rich	1			1		
**Source of drinking water**	Borehole	3.05	1.50–6.20	0.002	1.28	0.44–3.71	0.653
	Well	1.89	0.72–4.95	0.193	0.25	0.03–2.08	0.199
	Surface/rain/lake	1.86	0.88–3.92	0.105	1.05	0.32–3.48	0.934
	Other(water tank)	0.98	0.30–3.25	0.984	2.21	0.47–10.4	0.315
	Piped water	1			1		
**Education level**	Primary or less	2.85	1.51–5.35	0.001	0.82	0.25–2.69	0.749
	Secondary or more	1			1		
**Death Period**	2000–2006	12.96	8.29–20.26	<0.001	32.1	3.79–71.5	0.001
	2007–2014	1			1		
**Area**	Rural	6.05	1.91–19.18	0.002	0.53	0.22–1.31	0.170
	Semi-Urban	6.42	2.00–20.58	0.002			
	Urban	1					
**Number of deliveries**	None	1					
	Singletons	15.74	10.61–23.37	<0.001	6.76	2.70–16.9	<0.001
	Multiple deliveries	74.45	18.42–82.8	<0.001	-		
**Age(years)**	15–19	1			1		
	20–24	1.73	1.03–2.96	0.048	1.24	0.29–5.36	0.770
	25–29	2.41	1.44–4.04	0.001	2.61	0.67–10.1	0.166
	30–34	2.21	1.30–3.75	0.004	1.19	0.25–5.77	0.825
	35–39	1.37	0.74–2.53	0.315	1.38	0.26–7.30	0.705
	40–44	0.61	0.26–1.45	0.266	-		
	45–49	1.01	0.46–2.23	0.984	-		
**Distance to the nearest clinic(km)**	≥10	2.03	0.65–6.37	0.227			
	<10	1					
**HIV Status**	Positive	1.08	0.75–1.55	0.693			
	Negative	1					
**Parity**		1.02	0.94–1.09	0.684			

## Discussion

The study results has indicated a maternal mortality ratio of 650 per 100,000 live births, which shows why South Africa failed to achieve the 2015 MDG 5 target. Also, achieving less than 70 maternal deaths by 2030 still looks like a big ask for South Africa based on the current statistics. The multivariable analysis showed that socio-economic status, period of death and number of deliveries were significantly associated with maternal mortality. The expected hazard of maternal mortality was approximately four times higher in women from poor socio-economic statuses as compared to those from richer households. In addition, women with no children were at less risk as compared to those with one more children. The study also revealed that hazard risk of dying was very high before 2005 as compared to post 2005.

Past studies done in sub-Saharan Africa has shown that wealth index does influence maternal mortality. A study done in Malawi has shown that women from the rich wealth quintile had reduced odds of maternal mortality as compared to women in the poor wealth quintiles [17]. A similar study done in six sub-Saharan African countries namely, Ethiopia, Madagascar, Uganda, Zimbabwe, Zambia and Cameroon also showed that women from wealth households were less likely to experience maternal mortality as compared to women from poor households [18]. One of the reasons for high maternal mortality ratio in poor households is the inability of accessing maternal and reproductive health services as well as lack of transport to health care services [19, 20, 21, 22]. Similarly, a study done in Germany showed that the risk for maternal mortality was high in women with multiple births as opposed to those with singletons [23]. The reason being the fact that multiple pregnancy leads to a higher risk of problems for women such as pre-eclampsia, gestational diabetes and placental abruption [24].Studies in South Africa has indicated that mortality was generally high in South Africa before 2005. The main reason for that was because the Antiretroviral Therapy (ART) rollout was done post 2005 in many communities in South Africa [25]. Thus, before ART rollout mortality was largely high because of HIV since South Africa is among countries with high prevalence of HIV.

This study identified some factors influencing maternal mortality, namely socio-economic status, number of deliveries and period of death in rural South Africa. The identification of these factors will aid in improving the health and antenatal care of women with pregnancy related complications. Research has shown that wealth has an effect on the economic status of a household or individual [26]. It is believed that mothers from wealthier households have a high probability of attending first antenatal visits and subsequent visits as opposed to poorer mothers. In addition, household or individual wealth is positively associated with selecting a suitable health facility for delivery [27]. This is because usage of antenatal care services is influenced by the cost of the service and medication as well as other indirect costs like transport. As a result, it is expected that women from rich households’ would be able to use antenatal care services, as they may be able to pay for the costs associated with the service.More still the number of a woman”s deliveries does play role on maternal mortality as reported by Zeine et al.(2010) and Mekonnem et al.(2002) who found that the number of deliveries were associated with utilization of antenatal care services. In addition, health facilities need to provide well proficient and experienced birth attendants who are capable of handling maternal related issues and complications.

It is interesting to note that maternal deaths were significantly reduced after year 2006, which is the year when the ART rollout programme began in most communities. There is more cause for optimism to note the impact of the ART programme in scaling down maternal mortality, and this is even further supported by the insignificance of HIV status as a risk factor of maternal mortality. One can easily conclude that most HIV positive women are under the ART programme. However, it is important to highlight that studies have shown that Tuberculosis still remains the leading cause of death for women even those on treatment. It is very important for new research and policies to focus on co-ordinated efforts to treat HIV and TB. These findings will help guide and inform policy makers on key factors to target when addressing maternal mortality particularly in rural areas. Consequently, the field of public health and reproductive rights should priorities maternal mortality as a case that can be evaded and managed by health practitioners.

Statistics have shown that for the past two decades South Africa has made a substantial progress towards the reduction of maternal mortality. A recent report in South Africa has shown a decrease on maternal mortality from 189 per 100, 000 live births in 2009 to 134 per 100, 000 in 2016 [28]. The same trends have seen in this study population, with maternal mortality showing a decline from 600 per 100,000 live births in 2000 to 400 per 100,000 live births in 2014 [29].A report released by Statistics South Africa has indicated that HIV infection in pregnant women was still the major cause of maternal mortality in South Africa [30]. It is interesting to note that HIV status was not associated with maternal mortality in this study largely due to the huge rollout of the ART programme in this population. Despite the reduction in maternal mortality in South Africa, HIV infection is still major force, which contributes significantly towards maternal deaths.

One of the major strengths of this study is the huge population under follow-up in Africa Health Research Institute thorough capturing of vital population statistic such as migration, deaths and births over time. This enabled the control of selection bias that is mostly frequent in hospital-based researches, allowing rational study results, which are comparable to other study settings. The Africa Health Research Institute (AHRI) data set comprises of all maternal deaths that transpired within the study area regardless of the area of death, it covered deaths that occurred in hospitals, homes or elsewhere. In addition, the maternal mortality estimates from AHRI vary from the vital registration ones, largely due to the fact that the data on pregnancy related deaths is only completed from deaths of women aged 15–49 years, hence the need to upgrade the coverage of the vital registration system. The major limitation of this study is the verbal autopsy technique used to document the causes of deaths, this is likely to introduce response errors and misclassifications as well as recall bias. In addition, there were very few maternal deaths relative to the study population, which makes it difficult to monitor the evolution and trends of maternal mortality ratio.

## Conclusion

In conclusion, health policy interventions should focus on addressing the poor socio-economic status of women in rural areas as well as provided effective and efficient services for multi deliveries. It is very important that governmental interventions and policies put emphasis on these factors in rural areas, if the aim is to achieve the maternal mortality sustainable developmental target (SDG) in 2030.
